# Circulating Level of miR-378 Predicts Left Ventricular Hypertrophy in Patients with Aortic Stenosis

**DOI:** 10.1371/journal.pone.0105702

**Published:** 2014-08-26

**Authors:** Zhongxiu Chen, Chen Li, Yuanning Xu, Yajiao Li, Hao Yang, Li Rao

**Affiliations:** Department of Cardiology, West China Hospital of Sichuan University, Chengdu, Sichuan, China; University of Pittsburgh, United States of America

## Abstract

**Aims:**

Excessively high left ventricle mass is an independent predictor of adverse prognosis. MicroRNAs (miRs) play crucial roles in the regulation of left ventricle hypertrophy (LVH). However, few circulating miRs have been established as predictors of LVH in aortic stenosis (AS) patients. In this study, we aimed to investigate whether circulating levels of miR-1, miR-133, and miR-378 predict LVH in patients with AS.

**Methods and Results:**

One-hundred twelve patients with moderate to severe AS and 40 healthy controls were included in the study. Levels of miR-1, miR-133, and miR-378 in the plasma were measured by qPCR. Compared with healthy controls, AS patients had significantly lower circulating levels of miR-1, miR-133, and miR-378. AS patients with LVH had significantly lower miR-378 but not miR-1 and miR-133 compared with those without LVH. Linear regression analysis showed circulating miR-378 had strong correlation with left ventricular mass index (r = 0.283, p = 0.002) and logistic regression showed that lower miR-378 was an independent predictor for LVH in patients with AS (p = 0.037, OR 4.110, 95% CI 1.086 to 15.558).

**Conclusion:**

Circulating levels of miR-1, miR-133 and miR-378 were decreased in AS patients, and miR-378 predicts LVH independent of the pressure gradient. Further prospective investigations are needed to elucidate whether these circulating miRs affect clinical outcome.

## Introduction

Aortic stenosis (AS) is a common valve disorder that account for 43% of valvular heart diseases in adult patients [1]. In patient aged over 75, who have a high incidence of aortic valve calcification, the prevalence of hemodynamically significant (moderate to severe) AS can be as high as 2.8% [2]–[4]. Left ventricular hypertrophy (LVH) is the most common complication of AS. LVH was previously regarded as an adaptive remodeling. However, accumulating evidence shows excessively high LV mass is associated with lower LV systolic function and a higher rate of cardiovascular events, suggesting LVH is an independent predictor of adverse prognosis [5]–[8]. It was widely accepted that LVH was driven mainly by pressure overload. However, approximately 10–20% of the patients with critically severe AS do not have LVH. A recent study showed that the extent of LVH did not necessarily correlate with the severity of AS [9]. This evidence suggested that the pathogenesis and regulation of LVH was complicated and not fully understood yet. To further elucidate the regulation of LVH and find new risk factor predicting LVH in AS patients is thus of great importance.

Micro-RNAs (miRs) are non-coding RNA molecules of ∼22 nucleotides that function as a mechanism for negative post-transcriptional regulation [10]. MiRs plays central roles in the development of human diseases [11]–[15]. LVH is extensively controlled and regulated by miRs [16], [17]. Furthermore, miRs are surprisingly stable in blood, making circulating miRs attractive novel serum biomarkers [18], [19]. Thus, we hypothesized that circulating levels of some LVH-related miRs could be used as plasma biomarkers that predict LVH in patients with AS. In this study, we aimed to investigate the circulating levels of miRNA-1, miR-133, miR-378 in AS patients and their relationships with LVH.

## Materials and Methods

### Study Subjects

One-hundred twelve patients with moderate to severe AS (mean trans-aortic pressure gradient 20∼40 or >40 mmHg) [20] were included in the study. Patients with severe heart failure (NYHA class III to IV), severe combined mitral valve diseases or other structural heart diseases were excluded. Forty age-matched healthy controls were also recruited. The baseline characteristics of this patient cohort are shown in Table 1. The study protocol was approved by the Ethics Committee of the West China Hospital of Sichuan University (Sichuan, China). Written informed consent was obtained from all participants.

**Table 1:** Demographic and clinical characteristics of the patients.

	Controls(n = 40)	AS patients(n = 112)	P value
**Clinical characteristics**			
Age, (years)	53.3±10.8	53.3±10.5	0.988
Male, n (%)	23 (57.5%)	58 (51.8%)	0.507
SBP, (mmHg)	118.4±16.9	116.3±15.2	0.460
DBP, (mmHg)	70.7±10.5	70.4±9.7	0.848
HR, (bpm)	76.8±10.4	80.8±13.6	0.086
LVEF, (%)	60.3±5.6	62.3±10.9	0.284
LV mass index, (g/m2)	67.4±10.5	106.3±27.5	<0.001
**Etiology**			
Degenerative aortic valve diseases	/	46(41.1%)	/
Rheumatic valve diseases	/	53(47.3%)	/
Congenital bicuspid aortic vlave	/	13(11.6%)	/
**Medication**			
ACEI/ARBs, n (%)	/	28 (25.0%)	/
β-Blockers, n (%)	/	19 (17.0%)	/
Calcium antagonists, n (%)	/	7 (6.2%)	/
Diuretics, n (%)	/	47 (42.0%)	/

SBP: systolic blood pressure; DBP: diastolic blood pressure; HR: heart rate; LVEF: left ventricular ejection fraction; ACEI: angiotensin converting enzyme inhibitor; ARB: angiotensin receptor blocker.

### Echocardiography

Transthoracic echocardiography was carried out using an IE33 ultrasound system (Philips Medical Systems, Andover, MA, USA) equipped with a S5-1 transducer (Frequency 1.7–3.4 MHz). Interventricular septal thickness (IVSt), left ventricular diastolic diameter (LVDd) and left ventricular posterior wall thickness (LVPWt) were measured in the parasternal long-axis views at end-diastole. Three consecutive measurements were conducted to find the average value. Left ventricular mass (LVM) was calculated by the Deiereux correction formula: LVM(g) = 0.8×10.4 [(IVSt+LVPWt+LVDd)^3^-LVDd^3^]+0.6. Then, LV mass divided by body surface area (BSA) equals the LV mass index (g/m^2^). The mean trans-aortic pressure gradient was calculated from the jet velocity using Bernoulli's equation in the apical view [21]. LV ejection fraction (EF) was measured using the biplane Simpson's method using images acquired in the apical four-chamber and apical two-chamber views.

### Collection and procession of specimen

To establish a relationship between LVH and hsa-miR-1, mmu-miR-133a and hsa-miR-378, EDTA-anticoagulated peripheral blood samples were drawn from the antecubital vein of all subjects. Fasting blood samples were collected using disposable vacuum collection needles and a K_2_EDTA-anticoagulated vacutainer (Rich Science Industry Co., Ltd., Chengdu, China) at 6–8 o'clock in the morning. From these whole-blood samples, plasma was obtained by centrifugation (10 min at 3,000* g* at 4°C, no brake) within 1 hour after collection. All plasma samples were stored at −80°C until further analysis.

### qPCR for miRNA detection

MiRs were extracted from plasma using a miRcute miRNA isolation kit (Tiangen Biotech Co., Ltd., Beijing, China) according to the manufacturer's instructions. cDNAs were synthesized using a miRcute miRNA First-Strand cDNA Synthesis kit (Tiangen) in an S1000TM Thermal Cycler (Bio-Rad, USA). The miRcute miRNA qPCR detection kit (SYBR Green) (Tiangen) was used to assess the expression of individual miRs. The primers used were purchased from Tiangen Biotech Co., Ltd., catalog numbers were CD201-0114 (hsa-miR-378), CD201-0003 (hsa-miR-1), CD202-0006 (mmu-miR-133a) and CD201-0145 (hsa-U6). PCR amplification was performed using a C1000TM Thermal Cycler (CFX96TM Rea-Time System, Bio-Rad, USA) under the following conditions: 94°C for 2 min followed by 45 cycles of 94°C for 20 sec and 60°C for 34 sec. The production of specific products was confirmed using melting curve analysis (60-95°C) at the end of the amplification cycle. All experiments were performed in triplicate for each miRNA to obtain Ct values, and a non-template control was included on the same plate. A small stably expressed housekeeping RNA molecule, hsa-U6, was included as an internal control to normalize gene expression levels. Data were analyzed using Bio-Rad CFX Manager software (Bio-Rad), and the relative expression (fold difference) of candidate genes was calculated using the 2^−ΔΔCt^ method.

### Statistical analysis

All statistical analyses were undertaken using SPSS version 17.0 (SPSS, Inc, Chicago, IL). Data are shown as the means ± standard deviation (SD) or median (25th and 75th IQR). Statistical significance was assumed at p<0.05. Independent-samples t tests were used for baseline characteristics comparisons between the AS and control groups. Differences in three circulating miRs between the AS and control groups were compared using the Wilcoxon test. Differences in three circulating miRs among sub-groups were compared using the Kruskal-Wallis test. The relationship between trans-aortic pressure gradient and LV mass index, and between the three circulating miRs and LV mass index were evaluated using linear regression analyses. Logistic regression analysis was used to identify predictors of LVH in AS patients.

## Results

The clinical and demographic characteristics of the study population are shown in Table 1. There were no significant differences in age, sex, blood pressure, heart rate or LVEF between the two groups. The LV mass index was dramatically higher in AS patients than controls (106.3±27.5 vs. 67.4±10.5 g/m^2^, p<0.001). Among AS patients, 25.0% were taking ACEIs/ARBs, 17.0% were takingβ-blockers, 6.2% were taking calcium antagonists, and 42.0% were taking diuretics.

Fifty-four out of the 112 AS patients had LVH (>105 g/m^2^ for male, >95 g/m^2^ for female) according to the ESC guideline [22]. The clinical and demographic characteristics were generally similar between two sub-groups, except that LVH sub-group had significantly higher LV mass and lower heart rate(Table 2).

**Table 2:** Demographic and clinical characteristics of the AS patients with and without LVH.

	AS patients(n = 112)		
	Non-LVH(n = 58)	LVH(n = 54)	P value
**Clinical characteristics**			
Age, (years)	52.3±10.3	54.4±10.7	0.286
Male, n (%)	28(48.3%)	29(53.7%)	0.566
SBP, (mmHg)	116.5±13.4	116.0±16.9	0.877
DBP, (mmHg)	71.8±11.5	69.6±9.4	0.263
HR, (bpm)	83.7±15.6	77.7±10.3	0.019
LVEF, (%)	63.2±8.4	61.2±13.1	0.330
LV mass index, (g/m2)	85.1±16.3	127.8±18.7	<0.001
**Etiology**			
Degenerative aortic valve diseases	22(37.9%)	24(44.4%)	0.484
Rheumatic valve diseases	29(50.0%)	24(44.4%)	0.556
Congenital bicuspid aortic vlave	5(8.6%)	8(14.8%)	0.306
**Medication**			
ACEI/ARBs, n (%)	15(25.9%)	13(24.1%)	0.827
β-Blockers, n (%)	8(13.8%)	11(20.4%)	0.354
Calciumant agonists, n (%)	5(8.6%)	2(3.7%)	0.494
Diuretics, n (%)	20(34.5%)	27(50.0%)	0.096

SBP: systolic blood pressure; DBP: diastolic blood pressure; HR: heart rate; LVEF: left ventricular ejection fraction; ACEI: angiotensin converting enzyme inhibitor; ARB: angiotensin receptor blocker.

### AS patients have lower miR-1, miR-133 and miR-378 in blood circulation

**Figure 1:**
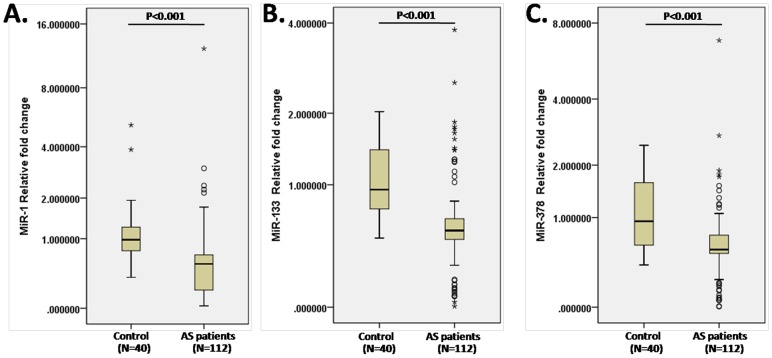
Circulating levels of miR-1, miR-133 and miR-378 in AS patients and controls. A. Circulating level of miR-1 in healthy controls and patients with aortic stenosis. B. Circulating level of miR-133 in healthy controls and patients with aortic stenosis. C. Circulating level of miR-378 in healthy controls and patients with aortic stenosis. The boxes represent the 25th, 50th, and 75th percentiles, while the whiskers represent the 10th and 90th percentiles.

Comparing with healthy controls, AS patients had significantly lower circulating levels of miR-1 (0.557 (0.202 to 0.711) vs. 0.979 (0.785 to 1.242), p<0.001), miR-133 (0.543 (0.469 to 0.651) vs. 0.947 (0.749 to 1.436), p<0.001) and miR-378 (0.560 (0.517 to 0.736) vs. 0.944 (0.627 to 1.610), p<0.001) (Figure 1).

### Low circulating miR-378 level predicts LV hypertrophy in AS patients

**Figure 2:**
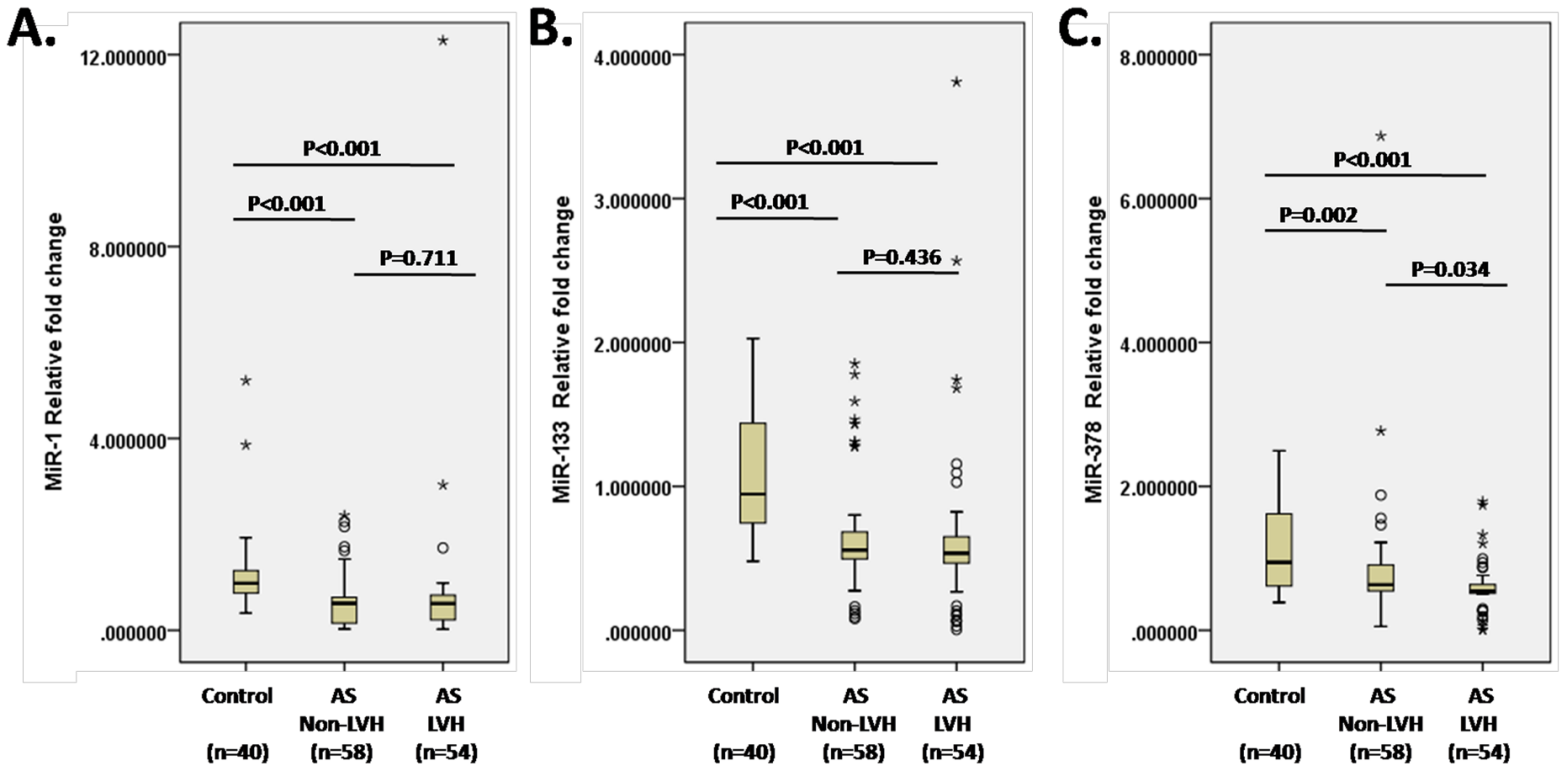
Circulating levels of miR-1, miR-133 and miR-378 in AS patients with and without LVH. A. Circulating level of miR-1 in healthy controls and AS patients with or without LVH. B. Circulating level of miR-133 in healthy controls and AS patients with or without LVH. C. Circulating level of miR-378 in healthy controls and AS patients with or without LVH. The boxes represent the 25th, 50th, and 75th percentiles, while the whiskers represent the 10th and 90th percentiles.

Significant decline of miR-1, miR-133 and miR-378 compared with healthy controls was observed in both AS sub-groups with or without LVH (Figure 2). However, patients with LVH had lower circulating level of miR-378 (0.539 (0.515 to 0.629) vs. 0.624 (0.549 to 0.903), p = 0.034) but not miR-1 (0.553 (0.287 to 0.730) vs. 0.551 (0.154 to 0.682), p = 0.711) or miR-133(0.532 (0.466 to 0.637) vs. 0.551 (0.498 to 0.676), p = 0.436) compared with AS patients without LVH.

**Figure 3:**
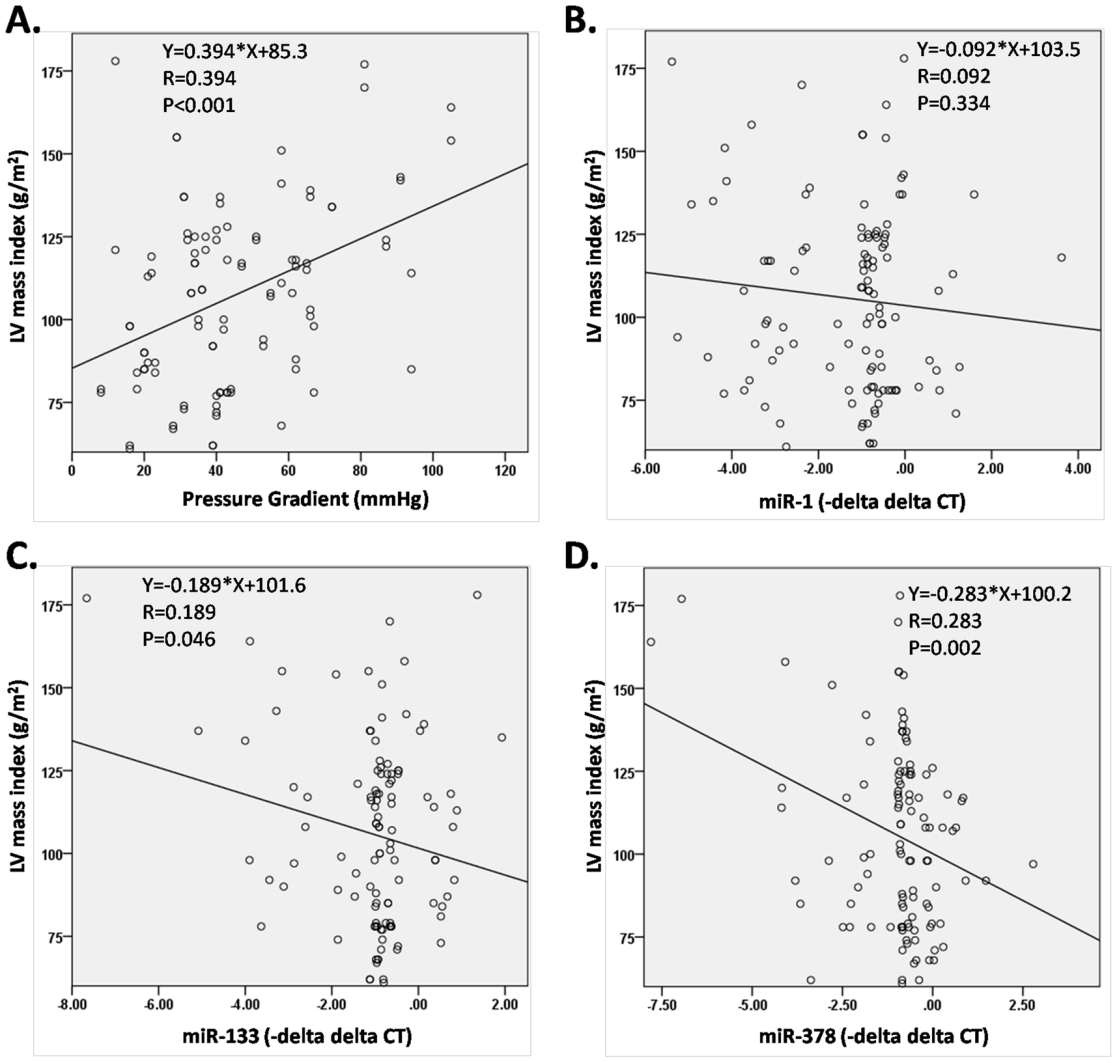
Trans-aortic pressure gradient and circulating level of miR-378 strongly correlated with LV mass. A. Linear regression of LV mass index and trans-aortic pressure gradient. B. Linear regression of LV mass index and circulating miR-1. C. Linear regression of LV mass index and circulating miR-133. D. Linear regression of LV mass index and circulating miR-378.

The linear regression analysis showed that LV mass index was closely correlated with trans-aortic pressure gradient (r = 0.394, p<0.001). As for the three circulating miRs, miR-378 showed the strongest correlation with LV mass index (r = 0.283, p = 0.002). MiR-133 had only a moderate negative correlation with LV mass index (r = 0.189, p = 0.046), while the level of miR-1 showed no significant correlation with LVH (r = 0.092, p = 0.334) (Figure 3).

In the muti-variant logistic regression analysis including all AS patients, a pressure gradient higher than 25 mmHg was the strongest predictor for LVH (LV mass index≥115 g/m^2^ for males or ≥95 g/m^2^ for females). Among the three miRs, only miR-378 lower than normal was identified as an independent predictor for LVH after adjusting for age, gender and pressure gradient (p = 0.037, OR 4.110, 95% CI 1.086 to 15.558)(Table 3.).

**Table 3:** Predictors of LVH in AS patients in the logistic regression model.

	Coefficient B	SE	P value	Adjusted OR(95%CI)
Age	0.026	0.020	0.202	1.026 (0.986 to 1.067)
Sex	−0.293	0.455	0.519	0.746 (0.306 to 1.818)
MiR-1 (lower than normal)	0.275	0.835	0.742	1.317(0.256 to 6.765)
MiR-133 (lower than normal)	−0.028	0.636	0.964	0.972(0.279 to 3.383)
MiR-378 (lower than normal)	1.413	0.679	0.037	4.110 (1.086 to 15.558)
Pressure Gradient (higher than 25 mmHg)	0.989	0.287	0.001	2.687 (1.533 to 4.713)

## Discussion

To date, over a dozen miRs have been identified to have a regulatory role in LVH, of which miR-1 and miR-133, derived from the same miRNA polycistron, are the most studied [16]. Both miR-133 and miR-1 are predominantly expressed in striated muscle and control myocyte proliferation and differentiation [23]. Alessandra et al. reported that miR-133 and miR-1 are down-regulated in both LVH animal models and patient samples [24]. Later studies demonstrated that silencing of miR-1 or miR-133 promotes LVH, while overexpressing them attenuates agonist-induced hypertrophy, confirming a causative role of miR-1 and miR-133 in LVH [24]–[30]. MiR-378 is a well-studied cancer-related miR that has been used in early-stage cancer detection [31]–[33]. MiR-378 is also enriched in the heart. MiR-378 regulats MAPK signaling, an important pathway in the pathogenesis of LVH, and loss of function study confirmed a causative role of miR-378 in LVH [34], [35]. Dysregulation of these miRs is important not only in molecular pathogenesis mechanisms, but also as potential novel biomarkers for LVH. A recent study trying to establish predictors of LVH regression after surgical valve replacement showed that miR-133 level, rather than increased aortic valve area or reduced mean transvalvular gradient, predicts regression of LVH [36]. This finding was confirmed in a larger cohort later. Importantly, the latter study reported that miR-133 in blood circulation is largely released from the heart, and the plasma level of miR-133 correlates well with its myocardial expression [37]. This finding was of great importance because direct measurement of cardiac miRs is not realistic in a routine clinical setting. In contrast, circulating miRs can be easily measured and thus potentially have wide clinical applications.

In our study, circulating levels of both miR-1 and miR-133 was significantly lower in AS patients. This finding is consistent with previous reports. Additionally, we report for the first time that circulating miR-378 was down-regulated in AS patients. Collectively, these results further support the idea that circulating levels of some cardiac-enriched miRs could serve as novel biomarkers for LVH. Furthermore, we found that circulating levels of miR-133 and miR-378 were negatively correlated with LV mass index in patients with AS. Multi-factor logistic regression analysis showed the negative correlation between miR-378 and LV mass was independent of the trans-aortic pressure gradient. LVH in AS patients is mainly driven by the excessive pressure overload. Accordingly, we found that the trans-aortic pressure gradient was the strongest predictor of LV mass index in AS patients. However, accumulating evidence shows that a complicated network of signaling pathways activated by pressure overload eventually control the cellular processes that contribute to LVH, and miRs play a pivotal role in regulating these LVH-related signaling pathways [16]. Our findings indicate that decreased circulating miR-378 could reflect a cardiac remodeling process initiated by the pressure overload and potentially determine the individual's vulnerability to the pressure overload induced by the AS.

Interestingly, miR-1 and miR-133, two well-studied LVH-related miRs, showed a weak or no correlation with LV mass index in AS patients. Two recent studies reported that miR-1 and miR-133 can be released into the circulation after cardiac injury due to prolonged aerobic exercise. In healthy marathon runners, miR-133a was positively correlated with the thickness of the intraventricular septum [38], [39]. In our study, we observed that some patients with extremely high LV mass had higher than normal circulating miR-1 and miR-133 (Figure 2.). It is possible that in some AS patients with severe LVH, miR-1 and miR-133 are increasingly released into the circulation from the hypertrophic and damaged heart, which offset the lower cardiac expression of miR-1 and miR-133.

Although the variation of circulating miR-378 level was associated with LVH in AS patients, in healthy controls, circulating miR-1, miR-133 and miR-378 levels showed no significant correlation with LV mass (data now shown). Moreover, we observed a relatively wide range of baseline circulating miR levels in the healthy controls. The clinical implications of these variations are still unclear. It is possible that lower miR-378 level in some healthy controls indicates they are more vulnerable to noxious stress and thus predict the risk of LVH in the future. This hypothesis should be tested in long-term follow-up studies.

In conclusion, the current study suggested circulating levels of miR-1, miR-133 and miR-378 were decreased in AS patients, and miR-378 predicts LVH independent of the pressure gradient. Further prospective investigations are needed to elucidate whether these circulating miRs affect clinical outcome.
